# Familial Generalized and Partial Lipodystrophies Due to Rare Biallelic Variants in *LMNA*

**DOI:** 10.3390/ijms27041724

**Published:** 2026-02-11

**Authors:** Michael Hwang, Charlita Worthy, Elaine Cochran, Megan Startzell, Ranganath Muniyappa, Chao Xing, Abhimanyu Garg, Rebecca J. Brown

**Affiliations:** 1 National Institute of Diabetes and Digestive and Kidney Diseases, National Institutes of Health, Bethesda, MD 20892, USAranganath.muniyappa@nih.gov (R.M.); 2 Eugene McDermott Center for Human Growth and Development, UT Southwestern Medical Center, Dallas, TX 75390, USA; 3 Lyda Hill Department of Bioinformatics, UT Southwestern Medical Center, Dallas, TX 75390, USA; 4 O’Donnell School of Public Health, UT Southwestern Medical Center, Dallas, TX 75390, USA; 5 Section of Nutrition and Metabolic Diseases, Division of Endocrinology, Department of Internal Medicine, Center for Human Nutrition, UT Southwestern Medical Center, Dallas, TX 75390, USA

**Keywords:** *LMNA*, lamin A, partial lipodystrophy, generalized lipodystrophy, compound heterozygous, homozygous, biallelic

## Abstract

Genetic lipodystrophies are a heterogeneous group of autosomal dominant and recessive disorders characterized by generalized or partial loss of body fat. Most patients with familial partial lipodystrophy (FPLD) have dominant inheritance with heterozygous pathogenic missense variants in *LMNA*. Here, we report two females with rare biallelic variants in *LMNA* presenting with divergent lipodystrophic phenotypes. Proband 1, a 32-year-old female, has near-generalized lipodystrophy (body fat 12.7%) due to compound heterozygous c.1745G>T (p.R582L) and c.1750C>T (p.R584C) *LMNA* variants. She was diagnosed with diabetes at age 17, hypertriglyceridemia at age 18, and metabolic dysfunction-associated steatotic liver disease (MASLD) at age 20. She was treated with metreleptin with only partial improvement in metabolic parameters. Her parents, heterozygous carriers of these variants, did not have lipodystrophy. Proband 2, a 35-year-old female, has partial lipodystrophy (body fat 21.2%) due to a homozygous c.1750C>T (p.R584C) *LMNA* variant. She was diagnosed with diabetes at age 19 and had a history of hypertriglyceridemia and mild hepatic steatosis. Her parents reportedly did not have lipodystrophy. These cases highlight the expression of *LMNA* variants in the homozygous or compound heterozygous state, manifesting in near-generalized and partial loss of body fat with distinct phenotypic heterogeneity.

## 1. Introduction

Lipodystrophy syndromes are a heterogenous group of rare genetic and acquired disorders characterized by the near-total or partial loss of adipose tissue in the absence of nutritional deprivation [1]. Among the genetic forms of lipodystrophy, the two most common subtypes are congenital generalized lipodystrophy (CGL) and familial partial lipodystrophy (FPLD), which differ in the distribution of fat loss, genetic etiology, and severity of associated metabolic complications [1].

CGL syndromes are a group of rare autosomal recessive disorders with variable phenotypes, but all marked by near-total fat loss apparent from birth or early infancy [2]. CGL is most frequently caused by biallelic pathogenic variants in *AGPAT2*, *BSCL2*, and more rarely in *CAV1* and *PTRF*/*CAVIN1* [2]. In addition to a near-total lack of adipose tissue, individuals with CGL exhibit prominent musculature, low serum leptin concentrations, and severe metabolic abnormalities [2]. These include severe insulin resistance, diabetes mellitus, hypertriglyceridemia, and metabolic dysfunction-associated steatotic liver disease (MASLD) [2]. Notably, leptin replacement therapy using recombinant human methionyl leptin (metreleptin) in patients with CGL results in dramatic improvements in many of these metabolic derangements [3].

In contrast, FPLD syndromes are a group of rare, usually autosomal dominant disorders, marked by partial fat loss that typically presents around puberty [4]. Fat loss predominantly affects the limbs and gluteal regions, while adipose tissue in the face, neck, and intra-abdominal areas is preserved and sometimes increased [4]. Because some fat depots are preserved in patients with FPLD, these patients have higher serum leptin concentrations compared to those with CGL and a correspondingly diminished therapeutic response to metreleptin [3]. FPLD is genetically heterogenous; those with autosomal dominant inheritance have heterozygous pathogenic variants in genes, including *LMNA* [5] and *PPARG* [6], and more rarely in *PLIN1* [7], *AKT2* [8], *ADRA2A* [9], *NOTCH3* [10], and *ACAA2* [11]. Those with autosomal recessive inheritance harbor biallelic pathogenic variants in *CIDEC* [12,13], *LIPE* [14], and *PCYT1A* [15].

*LMNA*, which encodes both lamin A (664 residues) and lamin C (572 residues), is the most frequently implicated gene in FPLD [16]. Lamins A and C are structural proteins of the nuclear lamina involved in maintaining nuclear stability, regulating chromatin organization, and modulating gene expression [16]. Most FPLD-associated *LMNA* variants are heterozygous and missense and occur in the C-terminal tail domain of lamin A/C, with p.R482Q and p.R482W being the most commonly reported [4,16]. While these heterozygous *LMNA* variants are classically associated with the “Dunnigan” FPLD phenotype, extremely rare cases of biallelic *LMNA* variants have been reported in individuals with more severe fat loss, suggesting a possible additive effect of *LMNA* variants on the severity of fat loss in lipodystrophy [17]. This phenotype is distinct from progeroid laminopathies, such as mandibuloacral dysplasia and Hutchinson–Gilford progeria syndrome, which also involve variants in *LMNA* and generalized fat loss but arise through different molecular mechanisms and exhibit distinct clinical features compared to CGL [4].

In this report, we describe two individuals with lipodystrophy due to biallelic variants in *LMNA*. Proband 1 is compound heterozygous for NM_170707.4:c.1745G>T (p.R582L; rs57830985) and NM_170707.4:c.1750C>T (p.R584C; rs578193315) and presented with near-generalized lipodystrophy, defined as missing essentially all metabolically active subcutaneous adipose tissue with minimal areas of preservation in small body regions (e.g., neck and genital regions). Proband 2 is homozygous for NM_170707.4:c.1750C>T (p.R584C) and presented with partial lipodystrophy, defined as missing metabolically active subcutaneous adipose tissue in large body areas (e.g., upper and lower extremities) with well-defined areas of preserved adipose tissue (e.g., head, neck, subaxillary, genital, and intra-abdominal).

## 2. Results

### 2.1. Proband 1

**Figure 1:**
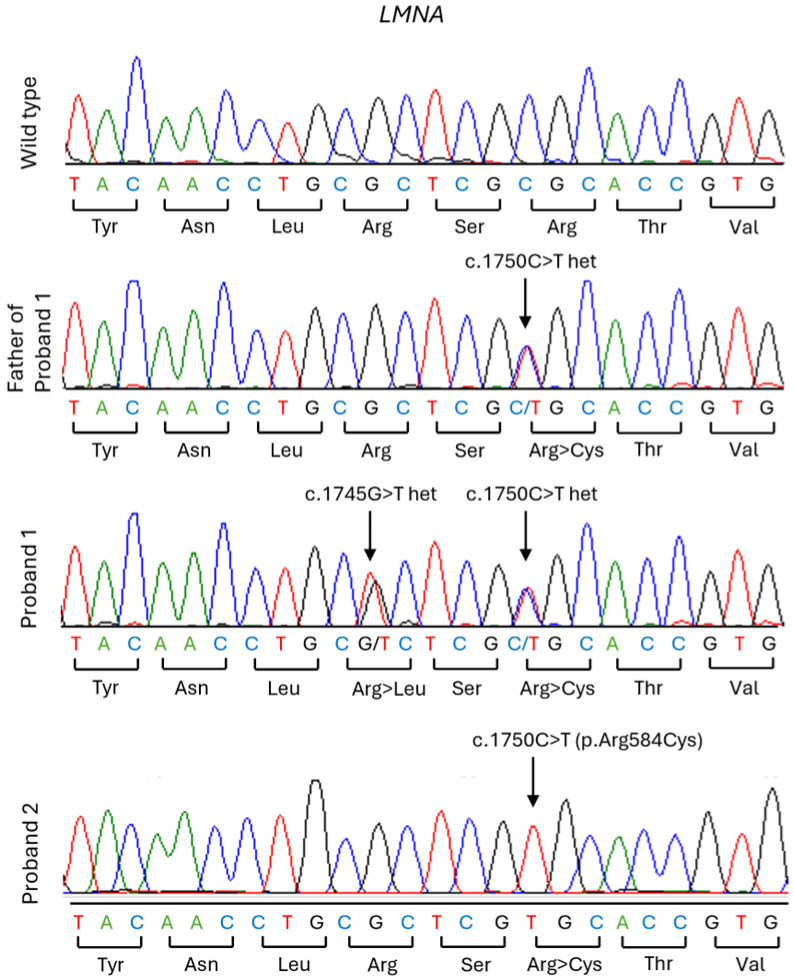
Sanger sequencing chromatograms showing *LMNA* variants in the probands and relative. Chromatograms show the wild-type sequence of *LMNA* exon 11, the heterozygous c.1750C>T (p.R584C) variant in the father of Proband 1, the compound heterozygous c.1745G>T (p.R582L) and c.1750C>T (p.R584C) variants in Proband 1, and the homozygous c.1750C>T (p.R584C) variant in Proband 2. The mother of Proband 1 carried the c. 1745G>T (p.R582L) heterozygous variant as per targeted next-generation sequencing; no chromatogram is available. Parents of Proband 2 did not undergo genetic testing.

Proband 1 is a 32-year-old Black/African American woman who was first given a diagnosis of CGL at age 17, after presenting with diabetes mellitus and an unusually muscular body habitus despite not being an athlete. Genetic testing was negative for variants in *AGPAT2* and *BSCL2* but revealed compound heterozygous c.1745G>T (p.R582L) and c.1750C>T (p.R584C) variants in *LMNA* (Figure 1; Table 1). She was subsequently referred to the National Institutes of Health (NIH) for further evaluation and management.

#### 2.1.1. Physical Examination and Anthropometric Measurements in Proband 1

Physical examination at initial NIH evaluation (age 20 years) revealed near-generalized fat loss with preservation of mechanical fat in the palms and soles. She displayed prominent musculature in the arms, legs, and abdomen, and did not appear to have any excess fat accumulation in the head, neck, or supraclavicular regions. Slight acanthosis nigricans was observed in the skinfolds of the posterior aspect of the neck and bilaterally in the axillae. Increased hair growth was noted on the chest, abdomen, and underneath the chin. Echocardiography at age 20 showed mild concentric hypertrophy of the left ventricle with hypertrabeculation of the left ventricular apex, and electrocardiography showed non-specific T-wave abnormalities.

**Figure 2:**
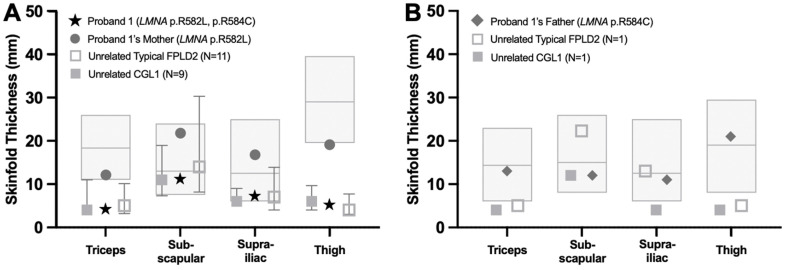
Skinfold thickness measurements of Proband 1 and her parents. (**A**) Skinfold thickness of Proband 1 (age 31 years) and her mother (age 67 years), represented by black stars and dark gray circles, respectively. The light gray bars show the 10th to 90th percentile values of normal adult females, with the median value marked by a horizontal line [20]. The median skinfold thickness measurements for unrelated adult females with CGL1 (N = 9) and typical FPLD2 (N = 11) are represented by gray, filled squares and gray, unfilled squares, respectively, with error bars showing the 10th to 90th percentile. (**B**) Skinfold thickness of Proband 1’s father (age 69 years) is marked by dark gray diamonds. The light gray bars show the 10th to 90th percentile values of unrelated normal adult males, with the median value marked by a horizontal line [21]. Measurements from an unrelated adult male with CGL1 (N = 1) and an unrelated adult male with typical FPLD2 (N = 1) are represented by gray, filled squares and gray, unfilled squares, respectively. Abbreviations: CGL1—congenital generalized lipodystrophy type 1; FPLD2—familial partial lipodystrophy type 2.

**Figure 3:**
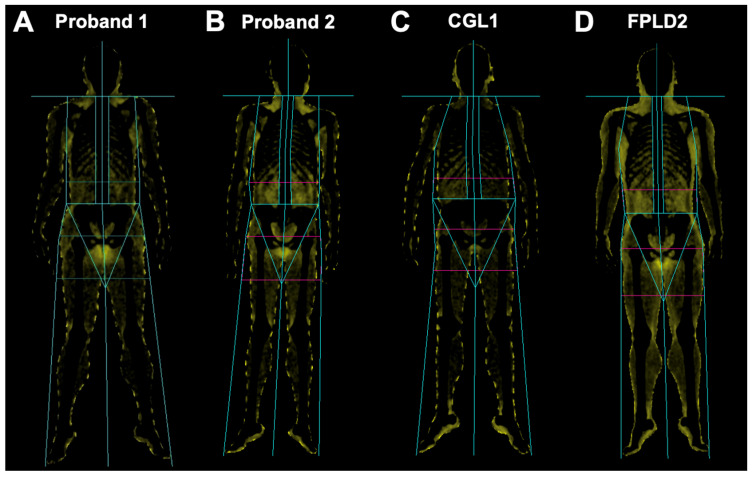
Comparison of whole-body fat distribution assessed by dual-energy X-ray absorptiometry (DEXA). Fat is shown in yellow, with all other tissues shown as black. Relative to sex-matched individuals with FPLD2 and CGL1, both probands display subcutaneous fat distribution at the lower end of the FPLD2 spectrum, with Proband 1 showing more severe depletion, approaching the fat distribution observed in CGL1. (**A**) Fat distribution of Proband 1 at age 32. (**B**) Fat distribution of Proband 2 at age 35. (**C**) Fat distribution of a 20-year-old female with CGL1 due to a homozygous *AGPAT2* (p.S100N [c.299G>A] and splice site c.493-1G>C) variant. (**D**) Fat distribution of an 18-year-old female with FPLD2 due to a heterozygous *LMNA* (p.R482W) pathogenic variant. Abbreviations: CGL1—congenital generalized lipodystrophy type 1; FPLD2—familial partial lipodystrophy type 2.

Her height was 164 cm, weight 71.1 kg, and body mass index (BMI) 26.4 kg/m^2^. Serum leptin was 1.7 ng/mL. Skinfold thickness was remarkably low across all measured sites in comparison to adult female normative data, with triceps and thigh thickness well-below the 10th percentile (Figure 2A). Dual-energy X-ray absorptiometry (DEXA) imaging done at age 32 showed near-total absence of subcutaneous adipose tissue in the proband, with minimal preservation of fat in the neck and genital region (Figure 3A). Total body fat percentage measured by DEXA was 12.7%, with regional fat percentages of 9.8% in the upper extremities, 8.9% in the lower extremities, and 15.3% in the trunk.

#### 2.1.2. Metabolic Complications of Lipodystrophy in Proband 1

Proband 1 was diagnosed with diabetes mellitus at age 17. She presented with polydipsia and weight loss with a random blood glucose level of 377 mg/dL with ketonuria, but she was not acidotic. Blood hemoglobin A1c (HbA1c) was 11%. She was initially treated with metformin (500 mg three times daily) and insulin (total daily dose 25 units). Despite this, glycemic control remained poor, and by age 19, her insulin requirements had escalated to over 90 units daily with blood HbA1c still over 10%.

At age 20, she was transitioned to U-500 insulin (total daily dose 40–100 units per day based on sliding scale), and her metformin dose was increased to 850 mg three times daily, which, within one month, decreased her HbA1c to 8.7%. Shortly thereafter, metreleptin (3.5 mg subcutaneously daily; 0.1 mg/kg/day) was initiated. Within six months, her HbA1c decreased to 6.5%, allowing for the discontinuation of insulin (Table 2).

Between ages 27 and 29, medication adherence declined. She reported missing 3–5 of 14 prescribed doses of metreleptin per week and missing other medications consistently. She re-established care at age 29, at which time her HbA1c was 10.2%. Metreleptin and U-500 insulin were resumed (total daily dose 106.5 units). Over the past 3 years, she has remained adherent to her medications, which has improved her diabetes control with HbA1c now ranging from 6.2 to 7.0%.

Hypertriglyceridemia was first documented at age 18 with a serum triglyceride level of 441 mg/dL, which was treated with atorvastatin (10 mg/day). After presenting to the NIH with a serum triglyceride level of 854 mg/dL, atorvastatin dosage was increased to 20 mg/day, and fenofibrate (145 mg/day) was started, which improved her triglycerides to 232 mg/dL within one month. Six months after initiation of metreleptin, her serum triglycerides increased to 474 mg/dL. At age 29, triglycerides were 616 mg/dL, which decreased to 147–254 mg/dL over the next 3 years after resuming consistent use of statin and fibrate. She denied ever having any xanthomas or xanthelasmas and had no history of pancreatitis.

Liver biopsy at age 20 showed steatohepatitis with moderate steatosis, moderate ballooning injury, moderate inflammation, and perisinusoidal fibrosis. Ultrasound of the liver showed the approximate liver span was 17–18 cm.

#### 2.1.3. Family History in Proband 1

Proband 1’s mother is a 67-year-old woman who was found to be a heterozygous carrier of the *LMNA* c.1745G>T (p.R582L) variant. She denied any history of abnormal fat loss, prominent musculature, or any other features suggestive of lipodystrophy during her childhood, puberty, or early adulthood. On examination, she had a BMI of 23.3 kg/m^2^ with normal fat distribution in the face, neck, trunk, and abdomen. She did appear to have lean upper and lower extremities, but there was no clear evidence of lipoatrophy. She did not exhibit acanthosis nigricans, hirsutism, xanthomas, or hepatomegaly. Total body fat percentage estimated based on skinfold measurements was 34.6%. Skinfold thickness was low-normal at the triceps and thigh (near the 10th percentile) but elevated at the subscapular and suprailiac sites relative to adult female normative data (Figure 2A). Laboratory tests revealed a new diagnosis of diabetes mellitus with an HbA1c of 7.9% (Table 2). Lipid profile showed elevated total cholesterol (266 mg/dL), mild hypertriglyceridemia (203 mg/dL), elevated LDL-C (183 mg/dL), and normal HDL-C (45 mg/dL). She reports that she was initially diagnosed with high cholesterol during her second pregnancy at age 37, but could not recall the age of diagnosis for hypertriglyceridemia. She denied any past medical history of pancreatitis or MASLD. Serum creatinine was elevated at 1.85 mg/dL with an estimated glomerular filtration rate of 30 mL/min/1.73 m^2^, consistent with stage 3b chronic kidney disease.

Proband 1’s father is a 69-year-old man who was found to be a heterozygous carrier of the *LMNA* c.1750C>T (p.R584C) variant. He has a past medical history of epilepsy (diagnosed at age 1) and coronary artery disease (diagnosed at age 63). He denied any history of abnormal fat loss, prominent musculature, or any other features suggestive of lipodystrophy during his childhood, puberty, or early adulthood. On examination, he had a BMI of 25.0 kg/m^2^ with normal fat distribution throughout his body, including the face, neck, trunk, and extremities. There was no obvious gain of fat in the abdomen, but increased abdominal girth was noted. He did not exhibit acanthosis nigricans, hirsutism, xanthomas, or hepatomegaly. Total body fat percentage estimated based on skinfold measurements was 21.4%. All skinfold thickness measurements were between the 10th and 90th percentiles (Figure 2B). Laboratory tests revealed very mild hypertriglyceridemia (153 mg/dL) and slightly elevated aspartate aminotransferase (35 U/L). He denied any prior diagnoses of hypertriglyceridemia, pancreatitis, MASLD, diabetes mellitus, or kidney disease.

### 2.2. Proband 2

We also identified an unrelated 35-year-old Black/African American woman with partial lipodystrophy carrying a homozygous c.1750C>T (p.R584C) variant in *LMNA* (Figure 1; Table 1).

She reported a muscular build beginning at the time of puberty. She reported that during periods of her life when she gained weight, fat accumulation was limited to her face and a small amount in her abdomen. Prior to her evaluation at NIH, she reported recent weight loss despite increasing appetite. She did not report any prior history of undernutrition, excessive physical activity, or childhood illness.

#### 2.2.1. Physical Examination and Anthropometric Measurements in Proband 2

Physical examination revealed partial fat loss. She exhibited an absence of subcutaneous fat in the extremities with prominent musculature. Subcutaneous fat was preserved but not increased in the trunk and abdomen, and excess fat deposition was present in her face, neck, and supraclavicular areas. Acanthosis nigricans was present bilaterally in the axillae. Echocardiography at age 35 showed mild dilation of the left atrium; an electrocardiogram was not performed.

Her height was 164 cm, weight 51.9 kg, and body mass index (BMI) 19.3 kg/m^2^. Total body fat percentage measured by DEXA was 21.2%, with regional fat percentages of 15.9% in the upper extremities, 14.7% in the lower extremities, and 26.5% in the trunk. DEXA imaging showed markedly reduced subcutaneous fat in the extremities, with preserved adipose tissue in the head and neck, visceral compartments, and genital regions (Figure 3B). Skinfold thickness was not measured. Serum leptin was 4.1 ng/mL.

#### 2.2.2. Metabolic Complications of Lipodystrophy in Proband 2

Proband 2 was diagnosed with diabetes mellitus at age 19, which was poorly controlled at the time of initial NIH evaluation with HbA1c of 11.1% while taking metformin (500 mg twice daily) and sitagliptin (100 mg daily).

She had a history of hypertriglyceridemia and mixed dyslipidemia with total cholesterol of 282 mg/dL, LDL-C 200 mg/dL, triglycerides 200 mg/dL, and HDL-C 41 mg/dL, which improved after treatment with niacin (500 mg daily). Lipid panel at the time of initial NIH evaluation is presented in Table 2. She denied ever having xanthomas or xanthelasmas and had no history of pancreatitis.

Abdominal ultrasound revealed mildly increased hepatic echogenicity in a patchy distribution, suggestive of mild hepatic steatosis. The liver and spleen were normal in size.

#### 2.2.3. Family History in Proband 2

Proband 2 denied any lipodystrophic features in any of her family members. Her mother is of Southeast Asian descent, and her father is of African descent. On review of photographs, neither her mother nor her sister appeared to have lipodystrophy. Her father had diabetes mellitus, but his body habitus was not known.

## 3. Discussion

We report two individuals with rare biallelic *LMNA* variants presenting with divergent lipodystrophic phenotypes. Proband 1, who had compound heterozygous p.R582L and p.R584C *LMNA* variants, was classified as having near-generalized lipodystrophy, while Proband 2, who had a homozygous p.R584C *LMNA* variant, was classified as having partial lipodystrophy. These cases highlight the possible synergistic effects of biallelic missense variants in *LMNA* on the severity of fat loss resulting in lipodystrophy and distinct phenotypic heterogeneity.

Proband 1 demonstrated anthropometric and clinical characteristics similar to CGL. She displayed near-total fat loss, with only preserved mechanical fat in the hands and feet. Supporting the clinical impression of CGL, skinfold thickness, total body fat percent by DEXA, and leptin levels were in ranges observed in patients with CGL, albeit at the upper end of typical CGL ranges. Furthermore, the severity of her metabolic complications of lipodystrophy was comparable to age-and-sex-matched patients with CGL1 (Table 2). However, despite these features resembling CGL, Proband 1 had a suboptimal response to metreleptin therapy. Both hypertriglyceridemia and insulin resistance worsened after 6 months on metreleptin. Notably, HbA1c improved from 8.7% to 6.5% after 6 months of metreleptin, although this improved glycemic control could have been due to concurrent intensification of insulin therapy (Table 2). Taken together, Proband 1’s phenotype closely mimicked CGL, yet her response to metreleptin was more typical of FPLD, highlighting the phenotypic overlap between these two conditions. Overlap of serum leptin and body fat percent among patients classified as having generalized and partial lipodystrophy has been shown previously [3]. Serum leptin level and body fat of Proband 1 fell at the upper end of published ranges for serum leptin and body fat for generalized lipodystrophy, and at the lower end for partial lipodystrophy. Interestingly, one patient reported in Diker Cohen et al. had even higher serum leptin (3.1 ng/mL) and body fat percent (16.4%) than Proband 1, despite a diagnosis of CGL type 1 due to *AGPAT2* pathogenic variants [3], highlighting the ambiguity of categorization between generalized and partial forms of lipodystrophy. In addition, assays for serum leptin, especially the RIA used to measure serum leptin in Proband 1, have high coefficients of variation and may have reduced precision in low ranges. Furthermore, cross-assay comparisons should be interpreted cautiously due to variability between RIA and ELISA [22].

By contrast, Proband 2 demonstrated anthropometric and clinical characteristics consistent with FPLD. She displayed regional loss of fat in the extremities and gluteal regions, which was first noted at the time of puberty. Fat was preserved in her trunk and abdomen and increased in her head and neck (Figure 3D). Her total body fat was 21.2%, which is higher than typically seen in generalized lipodystrophy but similar to values observed in subjects with FPLD, although her serum leptin was on the lower end at 4.1 ng/mL. Proband 2 also displayed less severe metabolic abnormalities compared to Proband 1.

The divergent phenotypes observed in these two individuals can be partially explained by their genotypes. The *LMNA* p.R582L heterozygous variant carried by Proband 1 was previously reported in a 44-year-old female with FPLD (subject UM28) [23]. She had a BMI of 28.2 kg/m^2^, serum leptin level of 4.6 ng/mL, and a past medical history of hepatic steatosis, but no prior diagnosis of diabetes mellitus, hypertriglyceridemia, dyslipidemia, or pancreatitis [23]. Unfortunately, details of her body fat distribution, including photographs, skinfold thickness measurements, DEXA, or MRI data, were not available. No other family members were included to study genotype–phenotype segregation. The heterozygous *LMNA* p.R582L variant has also been previously reported in a 45-year-old male with severe metabolic syndrome, but no lipodystrophy [24]. He had a BMI of 26.5 kg/m^2^ and total body fat of 31.3% [24]. Cellular studies from this individual showed increased nuclear misshaping, accelerated senescence rate, and reduced replication capacity, suggesting a likely pathogenic role of the *LMNA* p.R582L variant [24]. However, our Proband 1’s mother, who is also a heterozygous carrier for the p.R582L variant, did not exhibit overt lipodystrophy or severe metabolic abnormalities.

The *LMNA* p.R584C variant carried by both Proband 1 and Proband 2 has not been previously reported in the literature. Based on our observations, the p.R584C variant may act as a phenotype modifier, potentially exacerbating the severity of lipodystrophy when paired with another *LMNA* variant. Proband 1’s father, who is heterozygous (monoallelic) for p.R584C, showed no clinical evidence of lipodystrophy, and Proband 2’s parents, who are obligate heterozygotes, reportedly did not have clinical evidence of FPLD. In contrast, Proband 2, who is homozygous (biallelic) for p.R584C, displayed partial lipodystrophy. Furthermore, when p.R584C was inherited with another variant such as p.R582L, as seen in Proband 1, it was associated with a more severe, near-generalized lipodystrophy phenotype. Taken together, these observations suggest that p.R584C alone may be insufficient to cause lipodystrophy in the heterozygous state but could contribute to disease expression in the homozygous state or increase the severity of fat loss when combined with a pathogenic *LMNA* variant in a compound heterozygous fashion. Importantly, because family members of Proband 2 were not available for study, we cannot rule out the presence of a lipodystrophic phenotype in heterozygous family members. It should also be noted that these are only descriptive, hypothesis-generating observations from a small number of cases and that the precise role of the p.R584C variant remains unclear.

The notion that biallelic *LMNA* variants can produce more severe phenotypes than monoallelic variants in lipodystrophy syndromes is supported by the literature. Andre et al. [25] reported a large pedigree of 37 individuals from Reunion Island who all harbored the *LMNA* p.T655fsX49 variant, in either a heterozygous or homozygous state. They found that seven individuals with the homozygous variant had more severe fat loss, lower serum leptin levels, and a higher prevalence of metabolic abnormalities in comparison to those with the heterozygous variant [26]. Hegele et al. reported a New Brunswick family in which a 45-year-old female with FPLD due to compound heterozygous *LMNA* p.R482Q and p.V440M variants exhibited a severe lipodystrophic phenotype, including diabetes, dyslipidemia, and profound insulin resistance [27]. In contrast, relatives affected by single variants had less severe phenotypes, including her first cousin (heterozygous for p.R482Q), who had FPLD without metabolic complications, and her mother (heterozygous for p.V440M), who showed no features of lipodystrophy [27]. Similarly, Savage et al. reported three Caucasian siblings with FPLD due to compound heterozygous *LMNA* p.S583L and p.T528M variants [28]. These individuals had loss of fat in their extremities, with preserved visceral, abdominal, and facial fat [28]. However, their father and mother, who were heterozygous carriers of the p.S583L and p.T528M variants, respectively, were not clinically lipodystrophic [28]. Most recently, Soyaltin et al. reported a 29-year-old Turkish woman with a homozygous *LMNA* p.R582H variant who exhibited generalized fat loss, sparing only the mons pubis and genital region [17]. She had a lower serum leptin level and earlier onset of metabolic abnormalities compared to an unrelated individual with FPLD due to a heterozygous *LMNA* p.R582H variant [17]. This specific *LMNA* p.R582H variant has also been previously reported in a family with atypical FPLD [29,30].

The mechanisms by which compound heterozygous *LMNA* variants might give rise to a phenotype resembling generalized lipodystrophy are not established. However, similar gene dosage effects leading to increased severity of lipodystrophy have also been reported for *PPARG*, which can cause FPLD due to heterozygous pathogenic variants. Dyment et al. reported a 30-year-old female patient with CGL-like phenotype due to compound heterozygous *PPARG* p.E138VfsX168 and p.R164W variants [31]. She demonstrated generalized fat loss from infancy, severe hypertriglyceridemia leading to recurrent pancreatitis, and very low serum leptin levels [31]. In contrast, her father, who was heterozygous for the p.E138VfsX168 variant, was healthy aside from a muscular appearance and low serum leptin levels [31].

Importantly, these cases also highlight the clinical and genetic overlap between CGL and FPLD. Although traditionally viewed as distinct entities based on fat distribution, severity of metabolic abnormalities, and inheritance patterns, increasing evidence suggests that these conditions lie on a continuum. In Andrade et al., two individuals with monoallelic *LMNA* variants p.R541P and p.K486E were reported as having CGL [32]. Body fat was reported only in the individual with p.R541P, who had 22% body fat and preservation of head and neck fat, while the individual with p.K486E was described as having fat preservation only in the genital region [30]. Likewise, in Patni et al., two sisters with homozygous *LMNA* p.R545H variants were reported as having near-generalized fat loss [33]. The older sister (age 19 years) had 22.3% body fat with the absence of fat in the extremities and buttocks, minimal fat in the face and trunk, but preserved scalp, orbital, intra-abdominal, labial, and perirectal fat [33]. Serum leptin was 1.9 ng/mL [33]. The younger sister (age 17 years) displayed no subcutaneous fat in the extremities, but near-normal fat in the trunk and face [33]. Her total body fat was 26.8%, and serum leptin was 9.1 ng/mL [33]. Thus, the clinical distinction between generalized versus partial lipodystrophy can be ambiguous in patients with both monoallelic and biallelic *LMNA* variants. Additionally, Montenegro et al. reported a family where a 32-year-old mother and her two daughters (ages 12 and 8 years) all had the same homozygous p.R582C variant in *LMNA* but divergent phenotypes [34]. The mother had generalized lipodystrophy, while her daughters had partial lipodystrophy, highlighting the phenotypic heterogeneity of lipodystrophy syndromes [34]. Such heterogeneity among individuals with *LMNA* variants may reflect factors beyond the variant itself, such as genetic modifiers, epigenetic influences, environmental exposures, and stochastic developmental events.

The mechanisms by which variants in *LMNA* cause selective adipose tissue loss and concomitant prominent musculature remain incompletely defined, but recent studies have provided new insights. Corsa et al. demonstrated that adipocyte-specific *LMNA* knockout mice developed functional white and brown adipose depots postnatally that progressively disappeared around puberty [35]. In cell culture, primary mesenchymal precursors differentiated into adipocytes without impairment, but the resulting mature adipocytes exhibited increased lipolytic responses to adrenergic stimuli [35]. These findings indicate that lamin A/C is required for the long-term maintenance of adipose tissue rather than its initial development. Czapiewski et al. demonstrated that in preadipocytes from individuals with FPLD2, several myogenic loci fail to reposition to the repressive nuclear periphery during adipogenic differentiation [36]. Instead, these loci remained in transcriptionally active regions of the nucleus, leading to inappropriate persistence of myogenic gene activity at the expense of full adipogenic commitment [36]. This finding supports the hypothesis that, because muscle and adipose tissue share a common mesenchymal stem cell origin, impaired commitment toward adipogenesis in the setting of *LMNA* dysfunction may redirect precursors toward myogenesis, leading to the “pseudoathletic” appearance commonly described among individuals with *LMNA*-related lipodystrophy. Future studies are needed to clarify the precise mechanism by which *LMNA* variants contribute to fat loss and increased lean body mass.

In conclusion, these cases expand the known spectrum of *LMNA*-related lipodystrophy and illustrate that biallelic variants in *LMNA* can result in both near-generalized and partial fat loss. To our knowledge, this is the first report of near-generalized lipodystrophy arising from compound heterozygous *LMNA* variants and the first description of the p.R584C variant in association with lipodystrophy. Our findings emphasize the importance of including *LMNA* in the genetic evaluation of patients with phenotypes resembling generalized lipodystrophy, especially when testing for more common CGL-related genes, such as *AGPAT2* and *BSCL2*, is negative.

## 4. Materials and Methods

This study was approved by the institutional review board of the National Institutes of Health (study 76-DK-0006). Written informed consent was obtained from all subjects under this protocol, including the probands, their relatives, and the comparison cohorts with CGL and FPLD2 included in Table 2.

### 4.1. Laboratory Measurements

Blood samples were obtained after a 10 to 12 h overnight fast. Blood hemoglobin A1c and serum glucose, insulin, C-peptide, alanine transaminase (ALT), and aspartate aminotransferase (AST) were measured using standard techniques of the NIH Clinical Center Department of Laboratory Medicine. Total cholesterol, HDL-C, and triglycerides were measured on the Roche Cobas 6000 Analyzer (Basel, Switzerland). LDL-C was calculated using the Sampson-NIH equation [37]. Homeostatic model assessment of insulin resistance (HOMA-IR) was calculated as glucose (mg/dL) × insulin (µU/mL)/405.

Serum leptin was measured using commercial radioimmunoassay (RIA) in Proband 1 and enzyme-linked immunoassay (ELISA) in Proband 2 on fasting serum samples stored at −80 °C as previously reported [38]. The intra-assay and inter-assay coefficients of variation for the RIA kit (EMD Millipore, Billerica, MA, USA, catalog no. HL-81HK, RRID AB_2894698) were 9.29% and 9.96%, respectively. The intra-assay and inter-assay coefficients of variation for the ELISA kit (EMD Millipore, Billerica, MA, USA, catalog no. EZHL80SK, RRID AB_2894697) were 3.89% and 4.76%, respectively. All assays were performed per the manufacturer’s instructions.

### 4.2. Anthropometric Measurements

Height, weight, and body mass index (BMI) were determined using standard clinical methods. Skinfold thickness was measured with a +/− 1 mm Lange calipers at the truncal (suprailiac, subscapular) and peripheral (triceps, thigh) sites on the right side of the body. Total body fat percentage was determined either by whole-body dual-energy X-ray absorptiometry (DEXA; Hologic QDR 4500, Hologic, Bedford, MA, USA, or Lunar iDXA, GE Healthcare, Madison, WI, USA) or by using the Durnin/Womersley caliper method based on the skinfold measurements if a DEXA was not performed.

### 4.3. Genetic Testing

Sequencing of *LMNA* for Proband 1 and Proband 2 was performed at UT Southwestern as previously described [38]. The *LMNA* exons, including the splice site regions, were amplified in 11 segments from 50 ng of genomic DNA using the PCR and exon-specific primer pairs. The purified PCR products were sequenced using dye-terminator chemistry and an ABI 3730xl DNA analyzer. Sequence variants were verified by manually inspecting the chromatograms of both the wild-type and variant products. Genetic testing for Proband 1’s parents was performed by a commercial laboratory (Invitae, San Francisco, CA, USA).

## Figures and Tables

**Table 1:** Annotation of the two *LMNA* variants.

Genomic Location *	Reference	Alternative	Amino Acid Change *	Minor Allele Frequency in Populations of African Ancestry			Function Scores			Pathogenicity Classification **
				GnomAD	UK Biobank	All of Us	CADD	PolyPhen	GERP++	
chr1:156138534	G	T	R582L	0	0	0	28.2	0.977	2.05	Pathogenic
chr1:156138539	C	T	R584C	2.67 × 10^−5^	0	1.89 × 10^−5^	32	0.963	2.05	VUS

* The coordinates are based on hg38 and transcript NM_170707.4. ** Pathogenicity classification determined using Franklin by Qiagen (https://franklin.genoox.com/clinical-db/home, accessed on 4 February 2026). The Phred-like CADD score (−10*log_10_(percent)) assigns a score ranging from 1 to 99, with higher scores indicating a higher likelihood or disease-causing. For example, a score >20 indicates a variant is predicted to be in the top 1% of deleterious variants [18]. PolyPhen assigns a score between 0 and 1 to indicate the probability of a variant being pathogenic, with higher scores indicating a higher likelihood or disease-causing. The GERP score range is −12.3 to 6.7, with higher scores indicating a high constraint of sequences [19]. Abbreviations: CADD—combined annotation dependent depletion; GnomAD—genome aggregation database; PolyPhen—polymorphism phenotyping; GERP—genomic evolutionary rate profiling; VUS—variant of uncertain significance.

**Table 2:** Comparison of patient characteristics and clinical measurements.

	Optimal Range	Proband 1 (1 Month Pre-Leptin)	Proband 1 (Pre-Leptin Initiation)	Proband 1 (6 Months on Leptin)	Proband 1’s Mother	Proband 1’s Father	Proband 2	Females with CGL1 (N = 10)	Females with Typical FPLD2 (N = 7)
Genotype	N/A	Compound Heterozygous *LMNA* p.R582L, p.R584C			Heterozygous *LMNA* p.R582L	Heterozygous *LMNA* p.R584C	Homozygous *LMNA* p.R584C	*AGPAT2* (various genotypes)	*LMNA* (p.R482Q or p.R482W)
Age, years	N/A	20.2	20.3	20.8	67.8	69.3	35.8	20.3 ± 3.1	20.4 ± 2.2
BMI, kg/m^2^	18.5–24.9	26.4	25.5	23.8	23.3	25.0	19.3	22.8 ± 3.0	26.3 ± 3.0
Total cholesterol, mg/dL	<200	319	171	352	266	170	240	276 ± 106	172 ± 37
Triglycerides, mg/dL	<150	854	232	474	203	153	203	1333 (610, 3525)	267 (120, 497)
HDL-C, mg/dL	>40	39	34	41	45	65	41	30 ± 6 ^b^	26 ± 5
Insulin, µU/mL	2.6–24.9	54.7	43.0	97.1	15.5	16.6	16.0	34.7 (17.6, 85.8)	40.8 (21.2, 67.0)
Glucose, mg/dL	70–99	159	155	162	125	90	244	213 ± 99	90 ± 15
HOMA-IR	0–2	21.5	16.5	38.8	4.8	3.7	9.6	24.3 ± 15.5	10.6 ± 8.0
Hemoglobin A1c, %	4.0–6.0	10.2	8.7	6.5	5.0	5.0	11.1	9.7 ± 2.6	5.8 ± 0.7
C-Peptide, ng/mL	1.1–5.0	3.6	4.2	7.4	2.3	2.3	2.5	4.2 ± 2.4	4.9 ± 1.5
ALT, U/L	0–55	81	66	29	14	38	16	54 (43, 98) ^c^	32 (27, 64)
AST, U/L	5–34	45	40	33	14	35	15	35 (32, 78) ^c^	21 (19, 31)
Leptin, ng/mL	N/A	1.7	3.8	306.1	42.0	5.4	4.1	1.3 (0.9, 2.2)	8.1 (6.7, 14.7)
Total body fat, %	N/A	13.7	Not measured	Not measured	34.6	21.4	21.2	8.9 (8.2, 16.1) ^b^	24.2 (20.4, 27.7) ^a^

^a^ N = 6, ^b^ N = 7, ^c^ N = 9. Proband 1 is shown at her first NIH visit (1 month pre-leptin), and immediately prior to (pre-leptin initiation) and 6 months after metreleptin initiation. Values are compared to Proband 1’s parents, age-and-sex-matched individuals to Proband 1 with CGL1 and typical FPLD2 due to *LMNA* R482Q or R482W variants, and Proband 2. Both Proband 1 and Proband 2 were on exogenous insulin therapy at the time of measurement. Optimal clinical ranges are provided for reference. Group data are expressed as mean ± SD or median (25th–75th percentile) based on the data distribution. Homeostatic model assessment of insulin resistance (HOMA-IR) was calculated as glucose (mg/dL) × insulin (mcU/mL)/405. Total body fat was measured by skinfolds (parents of Proband 1) or by DEXA (all others). Leptin values were measured by either radioimmunoassay (RIA) or enzyme-linked immunosorbent assay (ELISA). Proband 1 and her parents were measured via RIA, while Proband 2 was measured via ELISA. In the FPL comparator cohort (N = 7), 3 subjects had leptin measured via RIA [8.1 (7.2, 8.4) ng/mL] and 4 via ELISA [7.8 (6.2, 15.8) ng/mL]. In the CGL comparator cohort (N = 10), 9 subjects had leptin measured via RIA [1.3 (0.93, 2.0) ng/mL] and 1 via ELISA (1.2 ng/mL). Abbreviations: ALT—alanine aminotransferase; AST—aspartate aminotransferase; CGL1—congenital generalized lipodystrophy type 1; FPLD2—familial partial lipodystrophy type 2; HDL-C, high density lipoprotein-cholesterol.

## Data Availability

All relevant data have been included in the manuscript. Further inquiries can be directed to the corresponding author.

## Abbreviations

The following abbreviations are used in this manuscript:

ALT	Alanine transaminase
AST	Aspartate aminotransferase
BMI	Body mass index
CADD	Combined annotation-dependent depletion
CGL	Congenital generalized lipodystrophy
DEXA	Dual-energy X-ray absorptiometry
ELISA	Enzyme-linked immunoassay
FPLD	Familial partial lipodystrophy
GERP	Genomic evolutionary rate profiling
GnomAD	Genome aggregation database
HbA1c	Hemoglobin A1c
HDL-C	High-density lipoprotein concentration
HOMA-IR	Homeostatic model assessment of insulin resistance
LDL-C	Low-density lipoprotein concentration
MASLD	Metabolic dysfunction-associated steatotic liver disease
NIH	National Institutes of Health
PolyPhen	Polymorphism phenotyping
RIA	Radioimmunoassay
VUS	Variant of unknown significance
